# Layout analysis of the RCEP international airline network based on hub identification using improved contribution matrix

**DOI:** 10.1038/s41598-024-61513-5

**Published:** 2024-05-12

**Authors:** Wendong Yang, Yulin Chi, Yining Huang, Wenbin Wei, Zhengjia Xu

**Affiliations:** 1 College of Civil Aviation, Nanjing University of Aeronautics and Astronautics, Nanjing, 211106 China; 2 Department of Aviation and Technology, San Jose State University, San Jose, CA 95192 USA; 3 School of Aerospace, Transport and Manufacturing, Cranfield University, Bedford, MK43 0AL UK

**Keywords:** Engineering, Aerospace engineering

## Abstract

The signing of the Regional Comprehensive Economic Partnership agreement brings new opportunities for the development of international air transportation. Faced with fierce competition, it is worth studying how hub airports should enhance competitiveness, and how low-cost carriers and full-service carriers should optimize the RCEP international airline network layout for better development. Aiming at providing suggestions for the development of hub airports, low-cost and full-service carriers in the RCEP international airline network, this paper identifies the hub airports, analyzes the layout of the RCEP international airline network, and the multi-layered characteristics based on an improved contribution matrix using data from 2010 to 2019 collected from the Official Airline Guide (OAG). This method comprehensively considers attributes of hub airports and the multi-layered characteristics of the airports and routes. The layout analysis indicates that the RCEP international transportation market presents a more open environment for competition and cooperation where base carriers are often the biggest supporters of hub construction. The multi-layered characteristics analysis reveals that low-cost carriers contribute more towards opening up new RCEP routes than full-service carriers. It is advised that carriers newly entering the RCEP international aviation transportation market and low-cost carriers dedicate to establishing new routes around their hub airports to monopolize this market and enhance their market share, whilst full-service carriers consolidate existing routes and increase route density to achieve economic benefits.

## Introduction

Air transportation has recently become an important way of carrying passengers and cargo within regional economies due to its distinctive benefits of high levels of internationalization and excellent long-distance accessibility^1^. Airline network is the primary factor to be considered in the development of air transportation because air transportation relies on networks to provide services and it determines the scale of carriers' transportation market, the economic benefits, and the allocation of aircraft, flights, and crews. Airline network layout is one of the most important steps during the design of airline networks due to its direct impact on transportation efficiency and economic benefits.

The signing of the Regional Comprehensive Economic Partnership (RCEP) agreement by ten ASEAN countries in 2020 has created new opportunities for the growth of carriers and international air transportation in these countries. On the one hand, the competitiveness of the hub airports and the overall layout of the RCEP international airline network need to be explored. On the other hand, given the fact that the market competition over carriers tends to be more intense with the expansion of the RCEP international airline network and the increasing number of carriers, it is worth studying how low-cost and full-service carriers should optimize the international RCEP airline network layout to improve competitiveness. Considering the traffic interaction between airports, the balance between safety and efficiency, and the coordinated interactive behavior of passengers and flights under big data^1^, the airline network is a complex system. Complex network theory is widely used in the analysis of complex networks, such as node importance evaluation^2,4^, layout structure analysis^1,5^, and network performance exploration^6,7^. Complex network theory in airline network research reveals the structure, and characteristics of airline networks by studying the connection patterns between nodes in the network, topology, and dynamic evolution laws. Therefore, this paper uses complex network theory to explore the construction of airline networks, the importance of airport nodes, and the analysis of airline network layout structure, which is an important foundation for the analysis of this article.

With the purpose of providing suggestions for the development of hub airports, low-cost and full-service carriers in the RCEP international airline network, this paper evaluates the importance of airports, identifies the hub airports, analyzes the overall layout of the RCEP international airline network, and comparatively analyzes carriers’ network layouts and multi-layered characteristics based on an improved contribution matrix using data from 2010 to 2019 collected from the Official Airline Guide (OAG). Evaluating the importance of airports and identifying the regional hubs of the RCEP international airline network is helpful for evaluating the competitiveness of hub airports and analyzing the network layout in the RCEP region. The comparative analysis of network layouts and multi-layered characteristics can provide references for low-cost carriers and full-service carriers to optimize their RCEP international airline network layouts and improve their competitiveness.

The main contributions of this paper are as follows:

1. This paper proposes a method for evaluating airport nodes’ importance and identifying hub airports in airline networks based on an improved contribution matrix, which comprehensively considers the weight of the network, the contribution of neighboring airports, and the multi-layered characteristics of airports compared to previous studies. The nodes are classified into four categories and the Susceptible-Infectious-Recovered (SIR) model is employed to assess the effectiveness of the proposed method and two other methods.
2. The competitiveness of hub airports and the overall layout of the RCEP international airline network are explored from 2010 to 2019. The layout of carriers’ international networks in the RCEP region and the multi-layered characteristics are further comparatively analyzed based on the multi-layered metrics.
3. Based on the analysis of the overall and carriers’ RCEP international airline network layout, and the multi-layered characteristics of airports and routes, suggestions for the development and optimization of hub airports, full-service carriers, and low-cost carriers in the RCEP international airline network are further provided.

The remainder of the paper is organized as follows. Section “Related works” reviews the most relevant paper about the two main methods of accessing the importance of airport nodes. Section “Methods and model” establishes a multi-layer regional airline network of different carriers based on the theory of multi-layer complex network, proposes an influential node identification method based on the improved contribution matrix, introducing the SIR model for validating method effectiveness, and multi-layer metrics used for analyzing the multi-layered characteristics of airports and routes. Section “Example analysis” accesses the importance of the airport nodes, classifies the airports into four categories, and verifies the rationality of the proposed methodology by SIR model. In the section “Layout analysis of the RCEP international airline network”, we identify the hubs, classify the airport nodes, analyze the layout of the RCEP international airline networks from overall and typical carriers’ perspectives, and explore the multi-layered characteristics of airports and routes. Section “Suggestions for airports and carriers” further provides suggestions for the development of the RCEP international airline network from the perspectives of airports and carriers. Finally, the results are concluded in the section “Conclusion”.

## Related works

Regional airline network hubs usually refer to important airports in a geographical area that connect different aviation markets, have convenient transit, and are densely populated with flights. Airline network layout refers to the coverage of routes, frequency, connectivity, and the selection of hub airports of carriers.

To analyze the airline network layout, previous studies have assessed the importance of airport nodes and classified airports. The methods of hub airport importance assessment and identification of previous studies are mainly classified into two categories: first, the classification of airport nodes using descriptive statistics and clustering models. Clustering models in airline network research groups or clusters nodes into subsets with similar characteristics or functions, which can help reveal the internal structure of the airline network, the patterns of interactions between nodes, and potential functional modules. Fuellhart et al. classified 131 airports in 53 global multiple airport regions and analyzed the role of these airports using hierarchical cluster analysis, utilizing supply-side variables such as aspects of competition, routes, and aircraft capacities^8^. Wang et al. constructed a weighted flight network for the Belt and Road Initiative, explored the clustering characteristics of national airports based on the Markov clustering algorithm, and analyzed the layout structure of the BRI flight network^9^. Rotondo developed a framework based on the strategic management and business models and used a cluster analysis on all 45 Italian commercial airports based on eight indicators reflecting dimensions of the business model framework^10^. Gao focused on a key bottleneck of outbound passenger movements in airport terminals, the security checkpoints, and used the Transportation Security Administration (TSA) Customer Throughput/Wait Times Reports to cluster hub airports of the United States^11^. Kim et al. proposed a generated delay concept to provide descriptive statistics to classify a target airport based on average propagated delays and average generated delays and performed delay propagation analysis for Korean domestic airports^12^. Chen et al. employed a multi-dimensional clustering to classify the airports of Chinese multi-airport regions based on competitive concentrations, the interaction between air transport and HSR, and airport community structure and further provided insights into Chinese airport functionality and impacts of the HSR network on the distribution of different types of airports^13^.

Since the regional airline network is a typical complex network, another useful way of identifying aviation hubs is by evaluating the influence and importance of airport nodes in airline networks using complex network theory. Zhou et al. proposed a method for determining key nodes in complex networks using importance evaluation matrices by defining node efficiency and node importance evaluation matrices^14^. Verma et al. classified airport nodes, and used the “t-core decomposition” method to divide the World Airline Network into three layers, and further revealed the hierarchical layout and resilience of the world airline network^15^. Du et al. classified airports based on the topological characteristics of airport nodes, and used the “k-core decomposition” method to divide the Chinese aviation network into three layers, and further analyzed the robustness of the Chinese aviation network^16^. Tu et al. constructed an importance evaluation matrix based on network edge weights and proposed a recognition algorithm based on proximity and importance evaluation matrices^17^. Wong et al. proposed a comprehensive world airport importance evaluation method and classified airports into five tiers, based on the centrality of neighboring airports, and further analyzed the layout of low-cost carriers' continents networks^18^. Xu et al. presented a novel signless-laplacian eigenvector centrality method to evaluate the centrality for both nodes and edges simultaneously by constructing a mutually updated iterative framework. Furthermore, tests were conducted based on several classical datasets including the US air transportation network and all produced satisfying results^19^. Du et al. proposed a novel method for identifying influential airports by defining three risk factors of an airport’s failure mode based on complex network theory and the transportation mechanism of airports in airline network flight flow. The Susceptible-Infected model is applied to evaluate the performance of the proposed method^20^. Yang et al. proposed an improved k-shell decomposition method to identify influential nodes, by highlighting the significance of the remaining network and neighboring nodes based on complex network theory. Experiments were conducted based on nine real datasets including the US air transportation network and seven classical topological metrics are compared to evaluate the performance of the proposed method^21^.

Table 1 summarizes the literature in terms of network type, network weight, and whether these papers considered the multi-layered characteristics of the network and the impact of neighboring nodes. Most studies focused on regional networks such as a certain country or continent, whilst the research on carrier networks nearly lacked investigation. In addition, most studies concentrated on the unweighted airline network structure, whereas the actual network is weighted. The weight is usually the number of flights and it impacts the accurate identification and classification of hub airports. Furthermore, few studies have considered the multi-layered characteristics of networks. Constructing a multi-layer airline network based on carriers can reveal the deeper internal relationships among them because a regional airline network consists of networks used by different carriers, which can provide carriers with more references when selecting airports, designing routes, and optimizing airline network layout^4^. Moreover, most studies neglected to consider the impact of neighboring airport nodes on the importance of a particular airport. The airline network consists of airport nodes and edges between airports. Airports do not exist independently in the network and there is passenger and cargo flow between airports. Therefore, assessing the importance of an airport requires considering the impact of neighboring airports.

**Table 1:** Review of related works.

Ref	Network type		Network weight		Multi-layered characteristics	Impact of neighboring nodes
	Regional network	Carrier network	Weighted	Unweighted		
Fuellhart et al. (2018)^8^	✓			✓		
Wang et al. (2019)^9^	✓		✓			
Rotondo (2019)^10^	✓			✓		
Gao (2020)^11^	✓			✓		
Kim et al. (2021)^12^	✓			✓		
Chen et al. (2022)^13^	✓			✓		
Zhou et al. (2012)^14^	✓		✓			✓
Verma et al. (2014)^15^	✓		✓		✓	
Du et al. (2016)^16^	✓		✓		✓	
Tu et al. (2017)^17^	✓		✓			✓
Wong et al. (2019)^18^	✓	✓		✓		✓
Xu et al. (2021)^19^	✓			✓		
Du et al. (2023)^20^	✓		✓			
Yang et al. (2024)^21^	✓			✓		✓
This paper	✓	✓	✓		✓	✓

This paper constructs weighted airline networks, identifies the hub airports, and analyzes the overall and the carriers’ RCEP international airline networks based on an improved contribution matrix, which considers the contribution of neighboring airports and the multi-layered characteristics of the airports and routes. At the same time, it analyzes the multi-layered characteristics of RCEP international airline networks to provide suggestions for the development of hub airports, full-service carriers, and low-cost carriers.

## Methods and model

**Figure 1:**
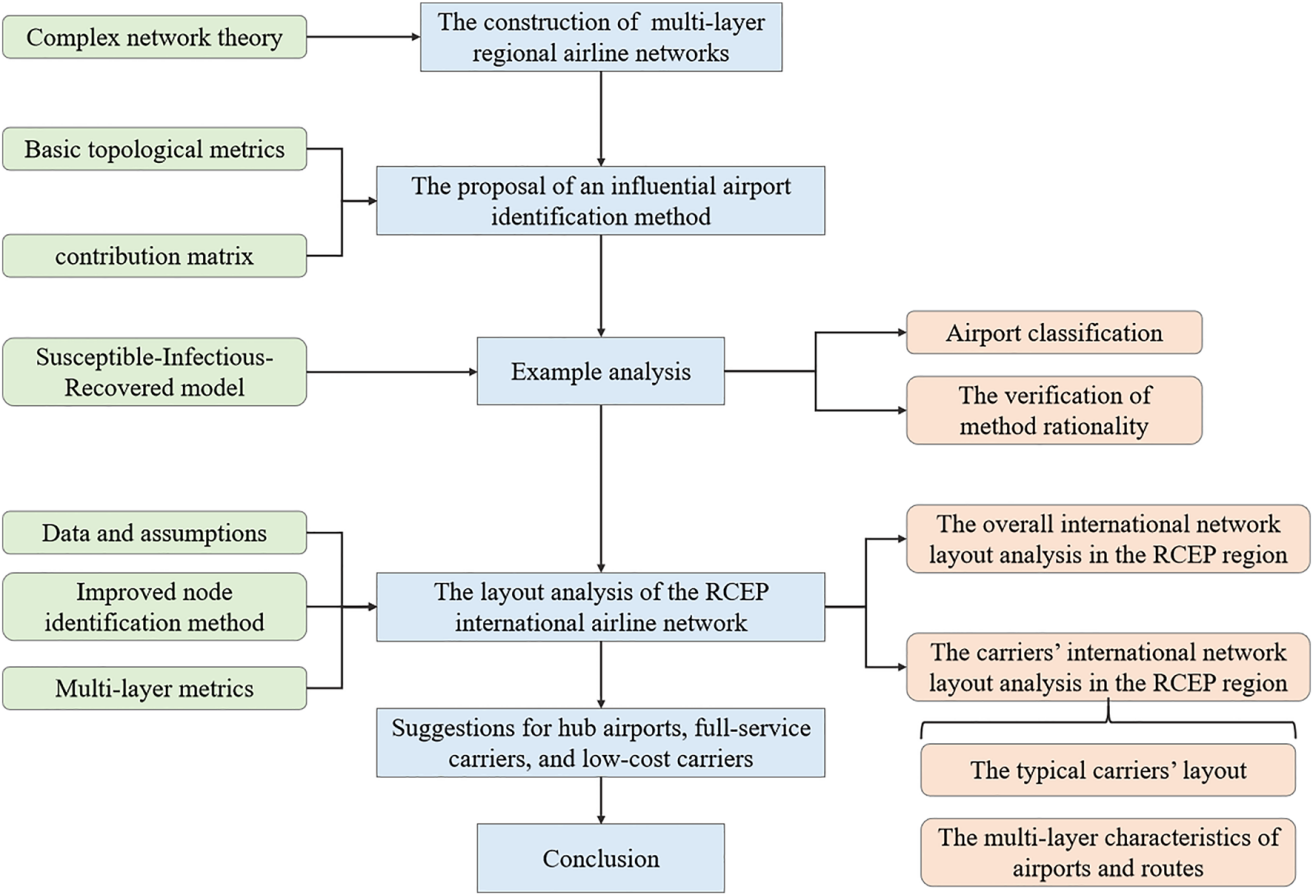
Mind map of this paper.

This section describes the models and metrics utilized for the analysis in this paper. Initially, the foundation of hub identification and airline network layout analysis—the construction of a multi-layer regional airline network is presented. Subsequently, the foundation of the influential node identification method is expounded, including the basic topological metrics and contribution matrix. Following that, the Susceptible-Infectious-Recovered (SIR) model is introduced, which is employed to verify the rationality of the proposed method. Lastly, the multi-layer metrics employed for the analysis of the multi-layered characteristics of airports and routes are displayed. On this basis, the overall layout of the RCEP international airline network is analyzed, and the comparative analysis of carriers' RCEP international airline network layout and its multi-layered characteristics is conducted to provide insights for the development of airports, full-service carriers, and low-cost carriers in the RCEP region. The mind map of this paper is shown in Fig. 1.

### Multi-layer regional airline networks construction

The construction of the multi-layer regional airline networks is the basis of the proposal of an influential airport identification method and the layout analysis of the RCEP international airline network. This paper mainly considers the direction and frequency of the routes and constructs a directed weighted regional airline network.

We first establish the single-layer directed weighted airline network of each carrier in the region, which uses the airport as the network node, the route connection as the directed edge, and the number of flights of the route during the observation period as the edge weight. Airline network is expressed as ${G^\alpha } = ({V^\alpha },\;{E^\alpha })$, where ${V^\alpha } = \left\{ {v_1^\alpha,v_2^\alpha, \ldots ,v_{N^\alpha }^\alpha } \right\}$ is the set of airport nodes and ${N^\alpha } = \left| {V^\alpha } \right|$ is the total number of nodes. ${E^\alpha } = \left\{ {e_1^\alpha ,e_2^\alpha , \ldots ,e_{M^\alpha }^\alpha } \right\}$ is the set of route edges, $e_{ij}^\alpha \in {E^\alpha }$ is the route edge from airport node $v_i^\alpha$ to $v_j^\alpha$ and ${M^\alpha } = \left| {E^\alpha } \right|$ is the total number of edges. Adjacency matrices ${A^\alpha } = \left( {a_{ij}^\alpha } \right)$ represents the directions of route connections of carrier $\alpha$ and weighted adjacency matrices ${W^\alpha } = \left( {w_{ij}^\alpha } \right)$ represents the number of flights on each route of carrier $\alpha$:$a_{ij}^\alpha = 1$ and $w_{ij}^\alpha$ equals the weight of $e_{ij}^\alpha$ if there is a flight from airport nodes $v_i^\alpha$ to $v_j^\alpha$, otherwise $a_{ij}^\alpha = 0$ and $w_{ij}^\alpha = 0$.

We then establish the regional multi-layer airline network based on the single-layer airline network of the carriers according to the multi-layer complex network theory^22^. If the regional airline network is composed of single-layer directed weighted airline networks of *H* airlines, the multi-layer airline network is represented as $HG = \left( {G,\;X} \right)$, where $G = \left\{ {{G^\alpha };\;\alpha \in \left\{ {1,2, \ldots ,H} \right\}} \right\}$ is a set of carriers' single-layer airline networks ${{\text{G}}^\alpha }\left( {{{\text{V}}^\alpha },\;{{\text{E}}^\alpha }} \right)$ and ${\text{X}} = \left\{ {{{\text{V}}^{\alpha ,\beta }} \subseteq {{\text{V}}^\alpha } \times {{\text{V}}^\beta };\;\alpha \ne \beta \;\;{\text{and}}\; \alpha ,\;\beta \in \left\{ {1,\;2, \ldots ,{\text{H}}} \right\}} \right\}$ is the set of cross-layer networks representing the correspondence of the same node in different airline network layers of different carriers. Adjacency matrix ${{\text{A}}^{\alpha ,\beta }} = \left( { x_i^{\alpha ,\beta }} \right)$ refers to the connectivity of airport nodes in different layers: $x_i^{\alpha ,\beta } = 1$ if $\alpha$-layer and $\beta$-layer have the same airport node ${v_i}$, otherwise $x_i^{\alpha ,\beta } = 0$.

The mapping approach enables the aggregation of single-layer airline networks of carriers in a regional airline network, thus simplifying the representation and analysis of multi-layer regional airline networks^21^. The region airline network can therefore also be expressed as $SG = \cup_{\alpha = 1}^H{G^\alpha } \left( { {V^\alpha },\;{E^\alpha } } \right) = \left( {V,\;E} \right)$, where $V = \left\{ {{v_1},{v_2}, \ldots ,{v_N}} \right\}$ is the set of airport nodes and $N = \left| V \right|$, and $E = \left\{ {{e_1},{e_2}, \ldots ,{e_M}\;} \right\}$ is the set of routes and $M = \left| E \right|$. Besides, adjacency matrices $A = \left( {{a_{ij}}} \right)$ and weighted adjacency matrices $W = \left( {{w_{ij}}} \right)$ represent the connectivity of the regional airline network: ${a_{ij}} = 1$ and ${w_{ij}} = \mathop \sum \nolimits_{\alpha = 1}^H w_{ij}^\alpha$ if $a_{ij}^\alpha = 1$, otherwise ${a_{ij}} = 0$ and ${w_{ij}} = 0$.

### An influential node identification method

To identify the hub airports of a regional airline network, we comprehensively discuss the basic topological metrics of airport nodes and propose an influential node identification method based on the improved contribution matrix, which considers the contribution of neighboring airports and the multi-layered characteristics of the airports. The Susceptible-Infectious-Recovered (SIR) model is introduced to verify the rationality of the proposed method.

#### Basic topological metrics

There are several basic topological metrics of nodes, which are used to explore the influence and importance of airport nodes in airline networks. The influence of airport nodes can be practically evaluated from both local and global perspectives. The local influence of the airport is mostly reflected in its transportation scale, which includes the number of airport routes and flights and can be represented by the indexes of node degree ${k_i}$ and node strength $W$. The global influence of the airport is primarily reflected in its hub role in airline networks, which can be expressed by the index of betweenness centrality $B{C_i}$.

Node degree ${k_i}$ is the number of directly connected node edges, which refers to the number of airport routes in the airline network and represents the breadth of airport connection:

1 $${k_i} = \mathop \sum \limits_{j \in N} {a_{ij}} + \mathop \sum \limits_{j \in N} {a_{ji}}.$$

Node strength ${W_i}$ is the sum of the directly connected edge weights of node $i$, which refers to the total number of flights in and out of the airport during the observation period:

2 $${W_i} = \mathop \sum \limits_{j \in N} {w_{ij}} + \mathop \sum \limits_{j \in N} {w_{ji}}.$$

Betweenness centrality $B{C_i}$ is the ratio of the number of times that it is passed through for the shortest path between any two nodes to the number of shortest paths between two nodes. The shortest path length in an airline network indicates the minimum number of flights required for passengers to travel from one airport to another. When an airport node lies in multiple shortest paths in the airline network, this indicates that the airport has a high circulation efficiency and a good transit function for passengers, and the airport can therefore have a great influence on the overall network operation:

3 $$B{C_i} = \mathop \sum \limits_{s,t \in N,i \ne j} \frac{{{l_{st}}\left( {v_i} \right)}}{{{l_{st}}}}$$

where ${l_{st}}$ is the total number of shortest paths from airport node ${v_s}$ to node ${v_t}$, and ${l_{st}}\left( {v_i} \right)$ is the number of shortest paths that passes through airport node ${v_i}$.

#### Importance measure based on the improved contribution matrix

Previous studies have found that the contribution matrix can be applied to identify key airport nodes in undirected airline networks by integrating airport location, route flow and other factors^17^. The contribution matrix concept is proposed based on the hypothesis that the value of a node can be evaluated by the importance of its neighboring nodes. The influence of the node on its neighboring nodes in an undirected network is proportional to the contribution of its degree to its neighboring nodes. Similarly, airports with direct flights in the same airline network usually depend on each other, so the importance of airport nodes is also closely related to the influence of their neighbouring airports. We can therefore transform the directed airline network into the undirected airline network to construct the traditional contribution matrix of airport nodes:

4 $$H = \left[ {\begin{array}{*{20}{c}} 1&{{\delta_{12}}\frac{{D_2}}{{{{\left\langle K \right\rangle }^2}}}}& \cdots &{{\delta_{1N}}\frac{{D_N}}{{{{\left\langle K \right\rangle }^2}}}} \\ {{\delta_{21}}\frac{{D_1}}{{{{\left\langle K \right\rangle }^2}}}}&1& \cdots &{{\delta_{2N}}\frac{{D_N}}{{{{\left\langle K \right\rangle }^2}}}} \\ \vdots & \vdots & \ddots & \vdots \\ {{\delta_{N1}}\frac{{D_1}}{{{{\left\langle K \right\rangle }^2}}}}&{{\delta_{N2}}\frac{{D_2}}{{{{\left\langle K \right\rangle }^2}}}}& \cdots &{1} \end{array}} \right].$$

where ${D_i}$ is the node degree of airport *i* in the airline network and $\left\langle K \right\rangle = \left( {\mathop \sum \limits_{i \in N} {D_i}} \right)/N$ is the average node degree of all airports. ${\delta_{ij}}$ is the node contribution allocation parameter, for which ${\delta_{ij}} = \;1$ if a flight link exists between airport nodes ${v_i}$ and ${v_j}$; otherwise ${\delta_{ij}} = 0$. ${H_{ij}}$ is the proportion of airport importance that airport node ${v_j}$ contributes to ${v_i}$. Diagonal elements refer to the contribution proportion of airport nodes to themselves, and hence equal 1.

However, the traditional contribution matrix only considers the node degree from a local perspective, causing a biased assessment of airport nodes’ influence. To make the evaluation more comprehensive, we improve the traditional contribution matrix by combining the metrics of node strength and betweenness centrality to weigh the important contribution proportion of airport nodes, which can maintain the directional characteristics of routes. The improved contribution matrix of airport nodes in the airline network is therefore expressed as:

5 $$WH = \left[ {\begin{array}{*{20}{c}} {{W_1}B{C_1}}&{{\delta_{12}}\frac{{{W_2}B{C_2}{D_2}}}{{{{\left\langle K \right\rangle }^2}}}}& \cdots &{{\delta_{1N}}\frac{{{W_N}B{C_N}{D_N}}}{{{{\left\langle K \right\rangle }^2}}}} \\ {{\delta_{21}}\frac{{ {W_1}B{C_1}{D_1}}}{{{{\left\langle K \right\rangle }^2}}}}&{{W_2}B{C_2}}& \cdots &{{\delta_{2N}}\frac{{{W_N}B{C_N}{D_N}}}{{{{\left\langle K \right\rangle }^2}}}} \\ \vdots & \vdots & \ddots & \vdots \\ {{\delta_{N1}}\frac{{{W_1}B{C_1}{D_1}}}{{{{\left\langle K \right\rangle }^2}}}}&{{\delta_{N2}}\frac{{{W_2}B{C_2}{D_2}}}{{{{\left\langle K \right\rangle }^2}}}}& \cdots &{{W_N}B{C_N}} \end{array}} \right]$$

where ${W_i}$ and $B{C_i}$ should be standardized according to the min–max method since they have quite different scales and dimensions.

Airport importance $W{D_i}$ in the airline network is defined as the sum of its own contribution and that of all its neighboring airports according to the improved contribution matrix $WH$:

6 $$W{D_i} = {W_i}B{C_i} + \mathop \sum \limits_{j \in N} \frac{{{\delta_{ij}}{W_j}B{C_j}{D_j}}}{{{{\left\langle K \right\rangle }^2}}} = OW{D_i}\; + \;NW{D_i}$$

where the contribution from the airport itself is defined as $OW{D_i} = {W_i}B{C_i}$, and the sum of the contributions from its neighboring airports is defined as $NW{D_i} = \mathop \sum \nolimits_{j \in N} {\delta_{ij}}{W_j}B{C_j}{D_j}/{\left\langle K \right\rangle^2}$.

Airport importance $W{D_i}$ concurrently evaluates the influence of the airport itself and its neighboring airports, which reflects the airport node importance relative to other airports in the airline network. The hub airport tends to have a stronger influence than its neighboring airports^17^. It is therefore assumed that airports themselves making greater contributions are more likely to be the hub airports of the airline network.

#### SIR model

The Susceptible-Infectious-Recovered (SIR) model has been extensively employed to assess the effectiveness of the node importance evaluation method and various centrality measures. The model classifies nodes into three categories: susceptible nodes, infected nodes, and immune nodes. At moment *t*, the number of susceptible nodes is denoted as $S\left( t \right)$, the number of infected nodes is denoted as $I\left( t \right)$, and the number of immune nodes is denoted as $R\left( t \right)$. The infected node $I$ infects a susceptible node $S$ with a probability of $\beta$. The infected node $I$ recovers as an immune node with a recovery probability of $\gamma$ and will not be infected again. The differential equations for the SIR model are as follows:

$$\left\{ {\begin{array}{*{20}{c}} {\frac{dS\left( t \right)}{{dt}} = - \beta S\left( t \right)I\left( t \right)} \\ {\frac{dI\left( t \right)}{{dt}} = \beta S\left( t \right)I\left( t \right) - \gamma I\left( t \right)} \\ {\frac{dR\left( t \right)}{{dt}} = \gamma I\left( t \right)} \end{array}} \right.$$

The influence of the initial set of infected nodes is measured by the number of the immune nodes $R$ in the network. In order not to lose the generality, this paper sets the recovery probability $\gamma = 1$. For each infected node, one randomly susceptible neighbor is infected with probability $\beta$ at each step. In weighted networks, node $i$ infects node $j$ with probability $\beta = {\left( {\frac{{{w_{ij}}}}{{w_M}}} \right)^\alpha }$^20^, where $\alpha$ is a positive constant and ${w_M}$ is the largest value of all. The faster the number of immune nodes increases in the network, the greater the influence of the initial set of infected nodes, and the greater the validity and effectiveness of the influential node identification method.

### Multi-layer metrics

Since the regional multi-layer airline network consists of routes used by different carriers, it is necessary to consider multi-layer metrics to analyze the attributes of airport nodes. This paper uses multi-layer complex network metrics, including node activity, multiplex participation coefficient, and route uniqueness to further explore the influence of the multi-layered characteristics of airports and routes and explore the relationship between carriers and airports.

In a multi-layer network, node ${v_i}$ is usually considered active in the α-layer network when its node degree $k_i^\alpha > 0$. Node activity ${B_i}$ is defined to specifically indicate the number of carriers operating at the airport:

7 $${B_i} = \mathop \sum \limits_{\alpha = 1}^H b_i^\alpha ,\;\;0 \leq {B_i} \leq H$$

where $b_i^\alpha = 1 - {\delta_{0,k_i^\alpha }}$. If node ${v_i}$ is active in the $\alpha$-layer network, then $b_i^\alpha = 1$; otherwise $b_i^\alpha = 0$. Node activity reflects the preference of carriers in choosing airport nodes, and can be used to determine the attractiveness of an airport to carriers and its relative importance in the airline network.

A high node degree of an airport in the single-layer airline network of a carrier suggests that the airport is vital for this carrier. However, it is worth noting that the importance of an airport can vary between different carriers. Multiplex participation coefficient ${P_i}$ can assess the heterogeneity of the edge distributions of nodes in different layers, so it is used to analyze the distribution of routes in the same airport for different carriers:

8 $${P_i} = \frac{H}{H - 1}\left[ {1 - \mathop \sum \limits_{\alpha = 1}^H {{\left( {\frac{k_i^\alpha }{{o_i}}} \right)}^2}} \right] \in \left[ {0,1} \right]$$

where ${o_i} = \mathop \sum \nolimits_{\alpha = 1}^H k_i^\alpha$ is the overlap degree of airport node ${v_i}$ in the multi-layer airline network. Multiplex participation coefficient quantifies the distribution of routes in each layer of the regional multi-layer airline network, characterizing the similarities and differences in route layouts at the same airport of different carriers. The airport nodes can be divided into three types according to ${P_i}$. The airport node belongs to the centralized type if ${P_i} \in \left[ {0,0.3} \right]$, the mixed type if ${P_i} \in \left( {0.3} \right.,\left. {0.6} \right]$ and the multi-layered type if ${P_i} \in \left( {0.6} \right.,\left. 1 \right]$. ${P_i} = 0$ indicates that all airport routes are operated by one carrier. In the contrast, ${P_i} = 1$ indicates that all carriers have the same route layout in the airport. Multiplex participation coefficient ${P_i}$ therefore also reflects the competition among carriers in the regional market, with a value closer to 1 indicating that competition is more intense.

The airline networks of different carriers may contain the same airport nodes, but the route layouts may be quite different due to the influence of the business model, transportation market, or air traffic rights policy. Route uniqueness indicates the proportion of routes in each carrier's single-layer airline network that do not appear in other layers of the regional multi-layer airline network, reflecting the difference and uniqueness of each carrier's route layout in the regional multi-layer airline network:

9 $${U^\alpha } = \frac{1}{{H^\alpha }}\mathop \sum \limits_{i,j} a_{ij}^\alpha \mathop \prod \limits_{\beta \ne \alpha } \left( {1 - a_{ij}^\beta } \right)$$

where ${U^\alpha }$ = 0 indicates that none of the routes are operated solely by carrier $\alpha$ in its airline network, and ${U^\alpha }$ = 1 means that all routes are operated solely by carrier $\alpha$ in its airline network. The larger the value of ${U^\alpha }$, the higher the route uniqueness of carrier $\alpha$.

## Example analysis

This section mainly assesses the node importance and classifies the nodes of an example network based on the proposed node importance assessment method, and uses the SIR model to compare the method proposed in this paper with the node importance assessment method in references 14 and 17 to validate the reasonableness of the proposed method, which lays the foundation for identifying the hubs of the RCEP international airline network and analyzing the network layout in the following section.

### Airport node classification

**Figure 2:**
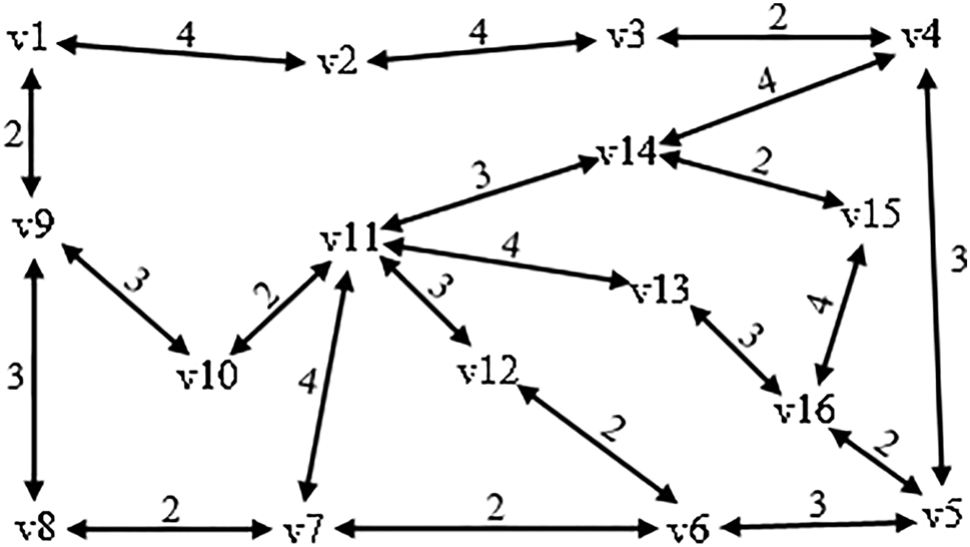
A randomly generated weighted topological network.

Routes and flights usually exist in both directions between two airports. We can simplify the calculation by taking a randomly generated weighted topological network as an example of airport node evaluation and classification while ignoring the difference of round-trip flights and directly providing the edge weights, as shown in Fig. 2.

The airport node evaluation method of the airline network based on the improved contribution matrix is used to calculate the importance of each node. The results and rankings are listed in Table 2. $OW{D_i}$, $NW{D_i}$, and $W{D_i}$ are the contribution of the airport itself, the contribution of neighboring airports, and the importance of the airport, respectively. It is found that although all airports are of similar importance, there are obvious distinctions between the contribution of each and those of its neighboring airports.

**Table 2:** Airport importance of the weighted topological network.

No.	Node	$OW{D_i}$	$NW{D_i}$	$W{D_i}$	Proportion of $OW{D_i}$ (%)
1	v11	1.000	0.129	1.129	88.6
2	v14	0.183	0.812	0.994	18.4
3	v7	0.099	0.746	0.845	11.8
4	v10	0.000	0.772	0.772	0.0
5	v13	0.023	0.748	0.771	2.9
6	v12	0.000	0.746	0.746	0.0
7	v4	0.198	0.133	0.331	59.8
8	v5	0.104	0.129	0.233	44.7
9	v6	0.047	0.089	0.136	34.6
10	v3	0.027	0.100	0.128	21.4
11	v9	0.107	0.005	0.112	95.3
12	v16	0.052	0.052	0.104	49.9
13	v15	0.000	0.102	0.102	0.0
14	v8	0.000	0.090	0.090	0.0
15	v1	0.018	0.061	0.079	22.9
16	v2	0.049	0.013	0.062	78.9

Based on the difference between the importance of the airport and the proportion of its contribution, the airport nodes can be categorized into four categories according to the definition of airport categories in the hub-and-spoke network:

1. Core-hub airport: Airport node v11 has the characteristics of high airport importance and a large proportion of the contribution. Although its neighboring airports have relatively small impacts on it, airport node v11 is still the most important airport in the airline network based on its significant scale and hub role. Airport nodes such as v11 are therefore regarded as core-hub airports.

2. Key-spoke airport: Airport nodes v14, v7, v10, v13, and v12 all have the characteristics of high airport importance but a small proportion of the contribution. These airports are connected to airport v11 by direct flights, which are near the center of the airline network and are the key departure locations or destinations for passengers, forming a transportation structure in which mainline and feeder-line routes cooperate to enhance transport efficiency. These airports are classified as key-spoke airports in the hub-and-spoke airline network.

3. Local-hub airport: Airport nodes v4, v5, v9, and v16 all have the characteristics of low airport importance but a large proportion of the contribution. These airports are less important than the core-hub and key-spoke airports throughout the airline network. However, their airport scale and hub role are clear in local areas of the airline network, and they play the same role as the core-hub airports in terms of structure. These airports are therefore considered as local-hub airports.

4. Feeder-line airport: v15 and v8 have the characteristics of relatively low airport importance and their own contribution is 0, which means their betweenness centrality is 0, and they do not play a hub role in the network for transshipment, thus they are classified as feeder-line airports. v1 has the characteristics of low airport importance and a small proportion of the contribution and is situated at the edge of the airline network. v2 has the characteristics of the lowest airport importance, which means that its own importance and the importance of its neighboring airports are comparatively small compared to the other airport nodes, and therefore it is also categorized as a feeder-line airport.

### Method rationality verification

This section first calculates the node importance of the example network using the proposed method, the method in references 14 and 17 and analyzes the difference in the ranking results. Furthermore, we compare and analyze the reasonableness of the proposed method using the SIR model.

The rankings of airport importance differ slightly because each method focuses on different aspects when assessing node importance. The airport importance calculation results and rankings of different methods are listed in Table 3. WD, WI and WP are the methods in this paper, reference 14 and 17 respectively. For example, v2 and v3 are similar in edge weight and location. The other two methods emphasize the difference in edge weights, which correspond to the flight frequency, leading to that v2 is more significant than v3. However, the proposed method suggests that carriers will consider the hub role of airport location the priority when choosing a new airport, rather than the flight frequency. When there is a small difference in edge weights between airports, v3 adjacent to local-hub airport v4 is more advantageous than v2 adjacent to feeder-line airport v1 for the carriers. Besides, v16 ranks high in the other two methods but low in this paper. Although v16 is identified as a local-hub airport, its importance is relatively low in the airline network since it is not in the center of the airline network and neither its airport scale nor distribution function has a substantial impact.

**Table 3:** Node importance ranking results for different methods.

No.	WD in this paper		WI in reference 14		WP in reference 17	
	Airport node	$W{D_i}$	Airport node	$W{D_i}$	Airport node	$W{D_i}$
1	v11	1.129	v11	0.412	v11	0.622
2	v14	0.994	v14	0.398	v14	0.512
3	v7	0.845	v16	0.378	v16	0.498
4	v10	0.772	v7	0.376	v7	0.445
5	v13	0.771	v12	0.366	v4	0.425
6	v12	0.746	v15	0.356	v12	0.402
7	v4	0.331	v4	0.355	v9	0.395
8	v5	0.233	v13	0.345	v15	0.394
9	v6	0.136	v2	0.342	v10	0.377
10	v3	0.128	v3	0.338	v13	0.374
11	v9	0.112	v10	0.322	v6	0.364
12	v16	0.104	v6	0.315	v5	0.355
13	v15	0.102	v5	0.295	v2	0.298
14	v8	0.090	v1	0.266	v1	0.256
15	v1	0.079	v9	0.242	v8	0.197
16	v2	0.062	v8	0.195	v3	0.128

**Figure 3:**
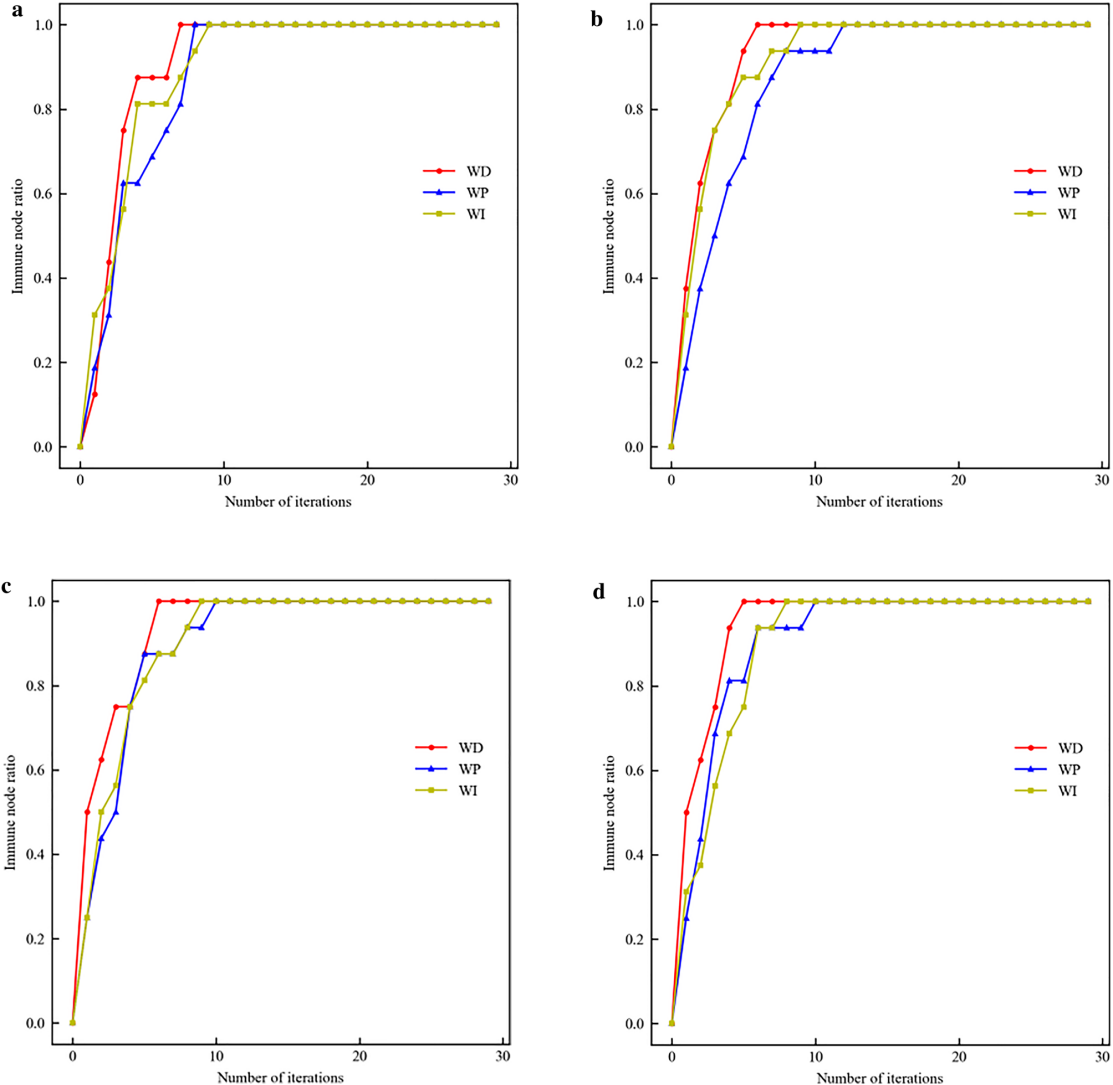
Node influence of different methods, different initial set of infected nodes.

The nodes in the top 3, 4, 5, and 6 of the importance ranking results of each method in Table 3 are used as the initial infected node set, respectively. The SIR model is run 100 times and the number of iterations is taken as 30, so as to compare the node influence of different initial infected node sets and the effectiveness of different methods. The faster the number of immune nodes increases, the higher the method's effectiveness. The results are shown in Fig. 3, with the horizontal axis denoting the number of iterations and the vertical axis denoting the node influence.

When the initial set of infected nodes contains 3 nodes, the node influence of WI rises faster than WD and WP when the number of iterations is 1. However, the influence of the set of nodes identified by WD is higher than that of the WP and WI methods overall. The same conclusion can be drawn when there are 4, 5, 6 nodes in the initial set of infected nodes. Therefore the effectiveness of the node influence assessment method proposed in this paper is comparatively verified. In Fig. 3a, WD needs 8 iterations to immunize all nodes, while in Fig. 3d, it can immunize all nodes after 6 iterations. This is attributed to the fact that the initial set of infected nodes in Fig. 3d contains 6 nodes, which can infect and immunize other nodes in the network more quickly.

## Layout analysis of the RCEP international airline network

This section identifies the hubs and classifies the airport nodes of the RCEP international airline network from 2010–2019 based on the proposed methodology. On this basis, the overall layout of the RCEP international airline network during the years 2010–2019 is analyzed and the comparative analysis of carriers’ RCEP international airline network layout and its multi-layered characteristics is conducted, which provides insights for the development of airports, full-service carriers, and low-cost carriers in the RCEP region in the following section.

### Data and assumptions

The data analyzed in this paper is obtained from the Official Airline Guide database (https://oag.cn/). We first collect the data on international flights in the RCEP region during 2010–2019 and construct the RCEP multi-layer international airline network. We then apply the methods to identify the regional aviation hubs of the RCEP and the multi-layer attributes of airport nodes. We make the following assumptions to better organize the analysis:

1. The regional routes from China to Hong Kong and Macao are considered international routes, but international routes for Taiwan are not considered.
2. The origin-to-destination and destination-to-origin directions are considered two routes.
3. Routes with stops are broken up into multiple routes comprising origin-to-stop and stop-to-destination legs since they involve one or more stop airports in addition to the arrival and departure airport.

### The overall layout of the RCEP international airline network

#### The overall layout of the RCEP international airline network in 2019

The top ten most important airports in the RCEP international airline network in 2019 are listed in Table 4. Airports including SIN, ICN, HKG, KUL, BKK, and PVG are found to be the core-hub airports of the RCEP international airline network since they all have the characteristics of high airport importance ($W{D_i} \geq$ 1.692) and a high proportion of the contribution ($\geq$ 6.8%). Each core-hub airport is also a large international airport located in a political or economic center of the country, with obvious advantages in air rights and geographical location, and abundant demand for passenger and cargo transportation. They could therefore be further considered the international aviation hub of the RCEP region. In short, the RCEP international airline network belongs to a multi-distribution and non-strict hub-and-spoke airline network with six regional aviation hubs since there are direct flights that connect these core-hub airports.

**Table 4:** Importance of the top ten airports in the RCEP international airline network in 2019.

No.	Airport node	$OW{D_i}$	$NW{D_i}$	$W{D_i}$	Proportion of $OW{D_i}$ (%)
1	SIN (Singapore Changi International Airport)	1.000	1.019	2.019	49.5
2	ICN (Incheon International Airport)	0.551	1.316	1.867	29.5
3	HKG (Hong Kong International Airport)	0.436	1.364	1.800	24.2
4	KUL (Kuala Lumpur International Airport)	0.312	1.481	1.793	17.4
5	BKK (Bangkok International Airport)	0.350	1.435	1.785	19.6
6	PVG (Shanghai Pudong International Airport)	0.115	1.577	1.692	6.8
7	KIX (Japan Kansai International Airport)	0.033	1.595	1.629	2.05
8	CAN (Guangzhou Baiyun International Airport)	0.013	1.612	1.626	0.82
9	PEK (Beijing Capital International Airport)	0.008	1.615	1.623	0.47
10	SYD (Sydney International Airport)	0.031	1.591	1.621	1.89

#### The evolution of the overall layout of the RCEP international airline network

**Figure 4:**
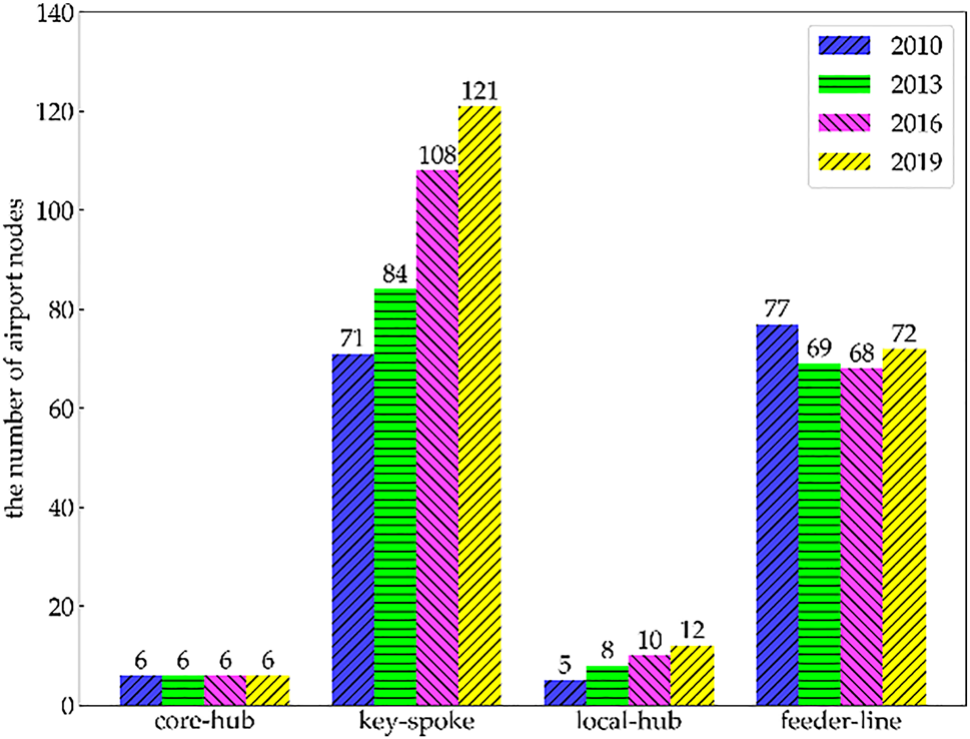
Classification of airport nodes in the RCEP international airline network from 2010 to 2019.

As can be seen in Fig. 4, the airports of the RCEP international airline network are divided into four categories according to the difference between the importance of the nodes and the proportion of the contribution. It is found that with the growth of the total number of airport nodes in the past 10 years, the number of core-hub airports has remained stable at six, whereas the numbers of key-spoke airports and local-hub airports have increased significantly and that of feeder-line airports has decreased slowly. It can be inferred that the core-hub airports of the RCEP international airline network are very stable, and the regional airline network is constantly expanding with the number of key-spoke airports around the core-hub airport increasing, which reflects the remarkable nature of the hub-and-spoke structure characteristics of the network.

As indicated in Table 5, we further research the airport development and layout evolution of the RCEP international airline network during 2010–2019. Table 5 has been sorted in descending order of node importance and lists the node contribution of itself. Although constituting a small proportion of the contribution, airports including BKK, KUL and PVG have been the top ten most important airports in the RCEP international airline network since 2010. The proportions of contribution made by airports BKK, KUL and PVG have shown an overall increasing trend, indicating that their own airport scale and hub role are constantly increasing, and their influence on the overall network is remarkable. In contrast, the proportions of the contribution made by airports PEK and SYD have fluctuated slightly over the past 10 years. These airports mostly rely on the influence of neighboring ones to maintain their importance, indicating that airports PEK and SYD have always been key-spoke airports of the RCEP international airline network. Although airports SIN, ICN and HKG were not among the ten most important airports in 2010 and 2013, they still rank high ($\geq$ 25/159) and represented a large proportion of the contribution ($\geq$ 23.01%). This indicates that they had already played the role of hubs in the overall RCEP international airline network, but their overall influences were not as high as that of airports BKK, KUL and HKG. Due to political economy, geographical location and scale advantages, the influences of these three airports and their neighboring airports have increased significantly within the regional airline network, so they can finally be ranked as the three most important airports after 2016 and have become solid core-hub airports.

**Table 5:** Airport importance ranking top 25 in the RCEP international airline network from 2010 to 2019.

No.	2010		2013		2016		2019	
	Airport node	Proportion of $OW{D_i}$ (%)	Airport node	Proportion of $OW{D_i}\;\;$ (%)	Airport node	Proportion of $OW{D_i}$ (%)	Airport node	Proportion of $OW{D_i}$ (%)
1	BKK	4.72	KUL	8.42	SIN	37.72	SIN	49.52
2	KUL	7.70	PVG	5.02	ICN	29.68	ICN	29.50
3	PVG	4.22	BKK	3.32	HKG	28.87	HKG	24.23
4	NRT	0.80	PEK	0.60	KUL	12.28	KUL	17.41
5	SYD	1.29	MNL	0.25	PVG	10.59	BKK	19.63
6	MNL	0.49	SYD	0.76	BKK	11.17	PVG	6.81
7	CAN	0.71	CAN	0.37	PEK	1.50	KIX	2.05
8	PEK	0.94	NRT	0.41	KIX	2.17	CAN	0.82
9	SGN	0.38	CGK	0.60	SGN	0.48	PEK	0.47
10	CGK	0.39	SGN	0.21	DPS	0.91	SYD	1.89
11	KIX	0.85	KIX	0.45	MNL	0.20	MNL	0.11
12	HKT	0.04	HKG	24.16	HAN	0.06	DPS	0.91
13	DPS	0.12	DPS	0.30	NRT	0.57	HAN	0.19
14	AKL	0.10	HAN	0.07	CGK	0.06	SGN	0.34
15	HAN	0.17	ICN	23.01	SYD	0.62	REP	1.88
16	SIN	28.83	MEL	0.26	CTU	0.06	CKG	0.09
17	MEL	0.29	HKT	0.02	REP	0.17	NRT	0.70
18	HKG	23.26	AKL	0.05	KMG	0.76	XIY	0.04
19	KMG	0.10	CTU	0.00	AKL	0.47	CTU	0.20
20	PNH	0.02	KMG	0.26	XIY	0.03	AKL	1.07
21	XMN	0.01	PNH	0.01	CSX	0.03	DAD	0.09
22	BNE	0.26	SIN	37.17	CKG	0.02	HKT	0.38
23	ICN	25.83	XMN	0.01	CNX	0.03	PNH	0.76
24	CRK	0.00	BNE	0.12	HKT	0.15	KMG	0.42
25	BKI	0.02	RGN	0.01	HND	0.11	XMN	0.02

The evolution of the core-hub airports also reflects the evolution of the RCEP international airline network layout. In 2010, the airline network mostly focused on the core-hub airports SIN, HKG and ICN with significant hub-and-spoke characteristics in the local area, and point-to-point structure characteristics in the overall area. But with the rapid development of the core-hub and key-spoke airports, the influence on the other airports has continuously increases. The global influence of the core-hub airports tended to be stable after 2016, which indicates that the RCEP international airline network is a stable multi-distribution and non-strict hub-and-spoke airline network.

### The layout of the carriers’ RCEP international airline network

#### The typical carriers’ layout of the RCEP international airline network

In the past decade, the RCEP international airline network size of various airlines has been continuously expanding, and the number of routes has significantly increased. The number of carriers with more than 100 routes has increased from 5 in 2010 to 24 in 2019. The top 20 carriers in terms of airline network size each year are shown in Table 6. Among the carriers with stopover routes in 2010, with the increase in the number of routes, the proportion of major carriers with stopover routes generally decreased significantly from 2016 to 2019, such as China Southern Airlines (CZ) and China Eastern Airlines (MU). Only a few carriers have seen an increase in the proportion of stopover routes, such as Spring Airlines (9C), which accounted for 18.6% of stopover routes in 2019, significantly higher than traditional full-service carriers. In 2010, carriers without non-stop routes added direct routes in the following ten years, such as Korean Airlines (KE) and Asian Airlines (OZ). Based on this, four typical carriers are selected to analyze their layouts in the RCEP international airline network: MU, the full-service carrier with stopover routes, OZ, the full-service carrier without stopover routes, 9C, low-cost carrier with stopover routes, and AK (Air Asia), low-cost carrier without stopover routes.

**Table 6:** Size of RCEP international airline networks of different carriers from 2010 to 2019 (top 20).

Rank	2010			2013			2016			2019		
	Carrier	Routes number	Stopover routes proportion (%)	Carrier	Routes number	Stopover routes proportion (%)	Carrier	Routes number	Stopover routes proportion (%)	Carrier	Routes number	Stopover routes proportion (%)
1	CZ	160	17.50	MU	221	20.81	MU	326	23.31	MU	375	13.87
2	MU	154	16.88	KE	160	0.00	CZ	254	15.75	CA	310	9.03
3	KE	146	0.00	CZ	141	18.44	KE	148	0.00	CZ	294	6.12
4	OZ	124	0.00	OZ	138	0.00	OZ	144	0.00	SQ	229	7.86
5	CA	112	30.36	CA	103	24.27	CA	142	14.08	NH	212	3.77
6	AK	92	0.00	AK	100	0.00	AK	128	0.00	JL	206	5.83
7	MH	91	18.68	VN	96	12.50	VN	122	4.92	OZ	190	0.00
8	TG	76	13.16	JQ	80	30.00	9C	119	10.92	QF	179	4.47
9	VN	76	18.42	MH	80	5.00	MI	98	18.37	KE	168	0.00
10	CX	71	28.17	MI	76	13.16	MF	92	32.61	VN	164	3.66
11	JQ	62	32.26	TG	74	5.41	KA	81	0.00	MF	161	14.91
12	MI	62	16.13	CX	69	26.09	TG	80	7.50	VA	159	3.77
13	JL	56	3.57	KA	66	0.00	NH	74	0.00	CX	148	6.76
14	NH	56	0.00	NH	64	0.00	FD	72	0.00	MI	141	9.93
15	NZ	52	1.92	5 J	62	0.00	5 J	70	0.00	EY	134	1.49
16	QF	52	11.54	JL	52	0.00	PR	70	5.71	AK	132	0.00
17	SQ	50	0.00	SQ	52	0.00	MH	68	2.94	9C	129	18.60
18	GA	48	16.67	QF	48	8.33	JQ	66	12.12	GA	124	3.23
19	PR	45	13.33	GA	46	8.70	CX	64	21.88	HU	120	6.67
20	KA	42	0.00	NX	46	0.00	TR	62	0.00	TG	117	3.42

The results of hub identification and layout evolution of the typical carriers’ RCEP international airline networks are obtained based on the proposed method. It is identified that the core hubs of MU in the RCEP international airline network in 2010 were PVG, HKG, and KMG, which are all located in China and overlap with the regional airline network’s two hubs. Their own contributions proportion are shown in Table 7. With the expansion of network scale, the core hub role of PVG is becoming increasingly significant, but the hub role of airport HKG and KMG is gradually decreasing. The overall network is centered around airport PVG, with airports HKG, KMG, and other regional hubs presenting a ‘one core, multiple zones’ layout structure distribution, gradually becoming more similar to the structural distribution of the regional airline network.

**Table 7:** The core-hub airports of the RCEP international airline network of typical carriers from 2013 to 2019.

Airlines	Airport name	2010 (%)	2013 (%)	2016 (%)	2019 (%)
MU	PVG (Shanghai Pudong International Airport)	61.58	59.86	73.86	81.42
	HKG (Hong Kong International Airport)	5.11	3.48	1.34	0.48
	KMG (Kunming Changshui International Airport)	1.27	2.62	3.65	2.20
OZ	ICN (Incheon International Airport)	99.25	99.35	99.46	98.63
	PUS (Gimhae International Airport)	49.81	59.11	42.60	80.11
AK	KUL (Kuala Lumpur International Airport)	93.06	94.97	96.78	96.27
	BKI (Kota Kinabalu International Airport)	2.09	2.75	16.69	23.32
9C	PVG (Shanghai Pudong International Airport)			58.18	72.83
	BKK (Suvarnabhumi Airport)			7.26	8.32
	KIX (Kansai International Airport)			4.38	2.39
	YTY (Yangtai International Airport)			0.27	18.39

From a topological analysis, the RCEP international airline network of OZ has core hub airports, including ICN and PUS, both located within South Korea and overlap with the regional airline network’s one hub. According to Table 7, the core hub role of airport ICN has remained significant in the past decade, while the size of airport PUS gradually increasing. The “dual-hub” layout of OZ in the RCEP international airline network is becoming more stable.

Similar to OZ, AK has core hub airports KUL and BKI in the RCEP international airline network, which are located within Malaysia, overlapping with the regional airline network’s one hub. In the past decade, the hub role of airport KUL has become increasingly stable, while airport BKI has gradually become a core hub airport since 2016 with the investment and construction of AK. The development of the RCEP regional economy and the expansion of airline markets have had a positive impact on each other.

Spring Airlines has many core hub airports, scattered throughout China, Japan, and Thailand. The core hub airport is mainly PVG, supplemented by BKK, KIX, and YTY, overlapping with the regional airline network’s two hubs. The hub role of airport BKK is slowly increasing, with a decrease in airport KIX and a significant increase in airport YTY, indicating that Spring Airlines intends to transfer its core hub from abroad to China.

Comparing the layouts and evolution of typical carriers' RCEP international airline networks, it is found that carriers often choose local airports in RCEP international aviation hubs as core hubs or base airports to develop and operate their airline networks, which is caused by political and traffic rights factors. In addition, the development of RCEP regional economies will also attract airlines to build and develop regional hub airports domestically, which is conducive to promoting the development of regional airline networks. The carriers' network and regional network have a positive impact on each other.

#### The activity of airport nodes

**Figure 5:**
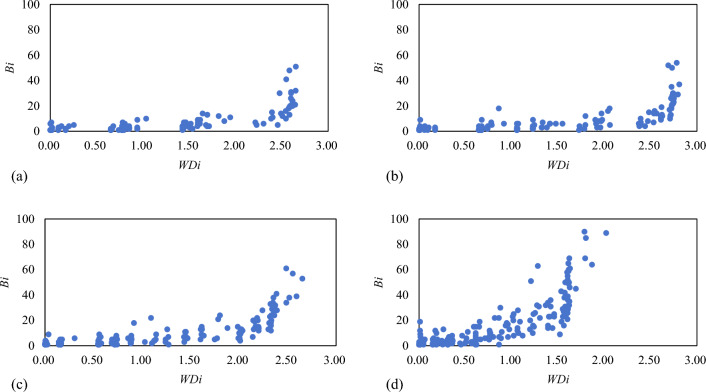
Relationship between airport importance and node activity from 2013 to 2019.

Figure 5 shows the relationship between airport importance and node activity in the RCEP international airline network over the past 10 years. Node activity positively correlated with airport importance, and airports of high importance tend to aggregate. This is mostly due to the influence of factors such as the economic situation and government-determined traffic rights. Carriers tend to choose large international airports in the political and economic centers of countries, which increases the importance of carriers operating flights in regional international aviation hubs. Besides, some airports have low airport importance but high node activity, such as Perth International Airport and Adelaide International Airport in Australia. This is mostly because carriers prefer to develop and operate routes around their base airports, which results in many local carriers competing for the transportation market of the few routes at the base airport. Last but not least, some airports are selected as bases or hubs only by low-cost carriers because full-service and low-cost carriers have different preferences in airline network layouts. There are therefore several airports exhibiting high airport importance but low node activity.

#### The multiplex participation coefficient of airport nodes

To compare the similarities and differences in the route layouts of carriers within the same airport node over the past 10 years, Table 8 lists the airport node types of the RCEP international airline network divided according to the multiplex participation coefficient. It is observed that the number and proportion of centralized and mixed airport nodes are gradually decreasing, while the number of multi-layered airport nodes has doubled in the past 10 years, and by 2019 accounted for 71.6% of the total number of airport nodes in the RCEP international airline network. This suggests that the continuous expansion of the RCEP international airline network has produced an evolutionary trend of airport nodes developing into the multi-layered type and most airports are in similar operating positions and provide similar transit services for passengers in routes used by different carriers. It can be inferred that carriers would follow the existing route layout structure in the RCEP international air transportation market when selecting airports or launching new routes, thus having similar international route layout structures. Additionally, 19.9% of airports in 2019 were centralized nodes, indicating that there are also many airports with routes monopolized by one or only a few carriers in the RCEP international airline network.

**Table 8:** Airport node types in the RCEP international airline network from 2013 to 2019.

Type	2010		2013		2016		2019	
	Number	Proportion (%)	Number	Proportion (%)	Number	Proportion (%)	Number	Proportion (%)
Centralized	52	32.7	44	26.3	41	21.4	42	19.9
Mixed	32	20.1	36	21.6	36	18.8	18	8.5
Multi-layered	75	47.2	87	52.1	115	59.9	151	71.6

**Figure 6:**
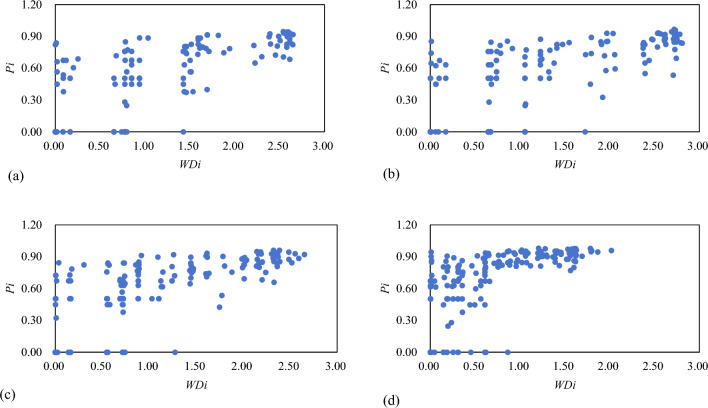
Relationship between airport importance and multiplex participation coefficient from 2013 to 2019.

Figure 6 shows the association between the multiplex participation coefficient and the importance of airports in the RCEP international airline network. The core-hub, key-spoke, and local-hub airports whose airport importance are greater than 1 are all considered multi-layered nodes, while the local-hub airports and feeder-line airports with airport importance of less than 1 could be considered mixed or centralized nodes. The findings suggest that the whole RCEP international air transportation market facilitates fair and open competition and a cooperative environment. However, the market and route competition is more fierce among hub airports of large importance because they usually attract more carriers. While feeder-line airports and new routes in the network have low influence, their development may increase in the future. Moreover, the number of airport nodes with high importance but zero multiplex participation coefficient has been decreasing in the past decade. In 2019, the multiplex participation coefficient of airport nodes with node importance greater than 1 was highly concentrated, indicating that the positioning and division of labor of each airport in the airline network are becoming increasingly clearer.

#### The uniqueness of route layout

**Figure 7:**
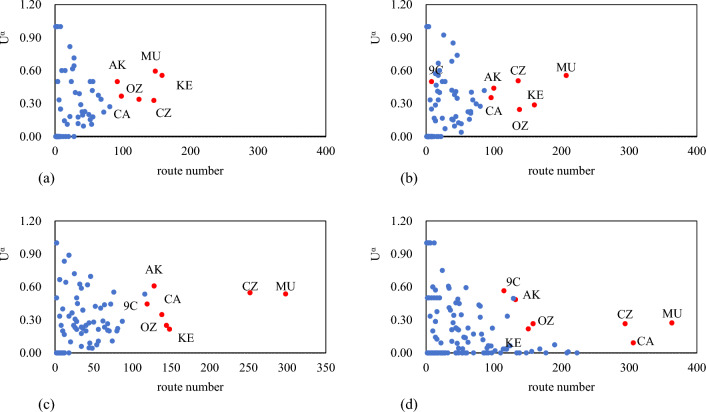
Relationship between route number and route uniqueness of typical carriers from 2013 to 2019.

The types, full names, and abbreviations of typical carriers are shown in Table 9. The relationship between the route uniqueness of typical airlines in the RCEP international airline network in the last decade and its number of routes is shown in Fig. 7. It is found that the route uniqueness of the airlines with the higher number of routes tends to be close to 0.5, indicating that the large airlines have about half of the routes independently operated and half of the routes competing in the RCEP international airline network. While most airlines have been experiencing steady growth in the number of route edges, several large full-service carriers had significantly lower route uniqueness in 2019.

**Table 9:** The types, full names, and abbreviations of typical carriers.

Types	Carrier	Carrier Code	Types	Carrier	Carrier Code
Full-service carrier	China Eastern Airlines	MU	Full-service carrier	Korean Air	KE
Full-service carrier	China Southern Airlines	CZ	Low-cost carrier	Spring Airlines	9C
Full-service carrier	Air China	CA	Low-cost carrier	Air Asia	AK
Full-service carrier	Asian Airlines	OZ

The relationship between the route uniqueness of typical airlines in the RCEP international airline network in the last decade and its number of routes is shown in Fig. 7. It is found that the route uniqueness of the airlines with the higher number of routes tends to be close to 0.5, indicating that the large airlines have about half of the routes independently operated and half of the routes competing in the RCEP international airline network. While most airlines have been experiencing steady growth in the number of route edges, several large full-service carriers had significantly lower route uniqueness in 2019.

The number of routes of full-service airlines MU, CZ, CA, OZ, and KE rank high and show an overall trend of growth, but the route uniqueness gradually decreases with the increase of the number of routes. This suggests that the new international routes in the RCEP region added by large full-service airlines after 2016 are mostly existing routes in the transportation market, and there are fewer new routes. Obviously, full-service airlines increased the frequency of flights on existing international routes, improved transportation efficiency in the RCEP international airline network, and gained benefits through transportation density economies.

Low-cost carriers have also strengthened their international airline networks in the RCEP region rapidly compared to full-service carriers. AK’s routes have increased 1.5 times over 10 years. 9C’s routes have increased 16 times from 2013 to 2019. The route uniqueness of AK and 9C has been maintained at 0.5 or below, indicating that low-cost carriers are more attentive to opening new routes than full-service carriers, and are adept at utilizing the scale effect economy to gain greater benefits by monopolizing the market of certain international routes in the RCEP region, and are the main force in the continuous expansion of the RCEP international airline network.

## Suggestions for airports and carriers

### Suggestions for airports development

Building an international aviation corridor will be an important measure to improve national competitiveness after the implementation of the RCEP agreement. It can be foreseen that competition among international aviation hubs in the RCEP region will also become more intense. At present, the hubs of the RCEP international airline network are distributed in Singapore, South Korea, Malaysia, Thailand, and China.

Firstly, stable aviation hubs such as Singapore Changi Airport, Kuala Lumpur International Airport, Incheon International Airport, and Hong Kong International Airport are recommended to maintain their central hub position and highlight their competitiveness in the international area. Secondly, Beijing Capital International Airport, Kansai International Airport, and Guangzhou Baiyun International Airport are international aviation hub preparations for the RCEP region and are important bases for carriers to build airline networks for the RCEP international market. Therefore, it is necessary to seize the opportunities of the implementation of the RCEP agreement to improve internationalization. Finally, based on hub identification, it is found that the main base carriers are often the biggest supporters and beneficiaries of hub construction. Therefore, it is necessary for airports to cooperate with carriers in the development of hub construction, maximize cooperation efficiency, help carriers expand their airline networks, and improve the convenience of transferring passengers and cargo.

### Suggestions for carriers' network layout planning

Although the RCEP agreement has stimulated and expanded the demand and market for international air transportation, the number of carriers participating in the RCEP international air transportation market is also increasing, and competition in the top market will become more intense with the expansion of the RCEP international airline network. This may also bring changes to the market structure and layout for the development of air transportation. Therefore, carriers' airline network layout planning must be more cautious.

According to the analysis of the size and evolution of the RCEP international airline network, it is found that the development of routes is mainly based on direct routes. However, the air transportation market is vast and the demand for outbound tourism is high. The development of stopover routes is not only beneficial for carriers to concentrate domestic transportation demand and form a certain scale economy effect but also for promoting the transformation of domestic transportation demand to international transportation demand, promoting a new development pattern of mutual promotion between domestic and international dual circulation in the air transportation industry, effectively improving circulation efficiency and reducing circulation costs. At the same time, based on the analysis of the multi-layered characteristics, most carriers have similar RCEP international airline layouts. When choosing transportation airports or adding new routes, they will pay attention to referring to existing routes. However, the competition for existing routes is intense, especially in aviation hubs and some base airports. In this case, considering stopover routes is beneficial for improving flight occupancy rates, and increasing the economic benefits and market competitiveness of carriers. Therefore, in the early stages of carriers' airline network development, carriers can build a certain proportion of stopover routes around base airports if hub competition is fierce.

Secondly, by analyzing the uniqueness of routes, it is found that low-cost carriers are more focused on opening up new RCEP routes than full-service carriers. The RCEP international airline network monopoly of large full-service carriers has reduced and increased the frequency of flights on existing international routes since 2016. This reflects the two strategies of carriers to expand their routes. One is to rely on opening new international routes in the RCEP region around the base airport and monopolizing this market, enhancing their market share in the regional international air transportation market. Another approach is to consolidate existing routes and increase flight frequency to achieve economic benefits. The former is suitable for new carriers and low-cost carriers entering the RCEP international air transportation market, while the latter is more suitable for large full-service carriers. However, while carriers continue to expand their routes and improve their hub layout, they also need to focus on building more reasonable flight schedules, fully leveraging the transfer and convergence role of hub structures, and improving the level of passenger and cargo transportation services.

## Conclusion

To analyze the layout of the RCEP international airline network and provide recommendations for the development of hub airports, full-service carriers, and low-cost carriers in the RCEP region, this paper identifies the regional aviation hub of the RCEP international airline network with the proposition of a method, which is based on an improved contribution matrix and comprehensively considers the contribution of neighboring airports and the multi-layered characteristics of the airports. The rationality of the proposed method is comparatively verified by the Susceptible-Infectious-Recovered (SIR) model. The proposed method is then used to identify the hub airports of the RCEP international airline network, and further analyze the layout of the RCEP international airline networks from overall and carriers’ perspectives and the multi-layered characteristics of airports and routes. Suggestions for the development of hub airports and the layout planning of full-service carriers and low-cost carriers in the RCEP international airline network are provided ultimately.

The following main results are obtained:

1. The RCEP international airline network is a multi-distribution and non-strict hub-and-spoke airline network with six regional aviation hubs.

2. The hub airport nodes with high importance in the RCEP international airline network have the characteristics of carrier aggregation and a few airports are monopolized by low-cost carriers. This indicates that carriers give priority to large international airports in political and economic centers of countries when choosing airports, and mostly operate routes around their base airports. The RCEP international transportation market presents a more open environment for competition and cooperation.

3. The results of hub identification reveal that the main base carriers are often the biggest supporters and beneficiaries of hub construction. Therefore, it is necessary for hub airports to seize the opportunities of the implementation of the RCEP agreement to improve internationalization, cooperate with carriers in the development of hub construction, and improve the convenience of transferring passengers and cargo.

4. The analysis of the multi-layered characteristics demonstrates that most carriers have similar RCEP international airline layouts and they tend to operate existing routes rather than open new routes. In this case, carriers are recommended to build a certain proportion of stopover routes around base airports to improve flight occupancy rates and increase the economic benefits and market competitiveness of carriers.

5. The analysis of the uniqueness of routes finds that low-cost carriers are more focused on opening up new RCEP routes than full-service carriers. Based on this, it is suggested that new carriers and low-cost carriers entering the RCEP international air transportation market open new international routes around the base airport to monopolize this market, and enhance their market share. Large full-service carriers can consolidate existing routes and increase flight frequency to achieve economic benefits. In addition, all carriers need to focus on building more reasonable flight schedules, fully leveraging the transfer and convergence role of hub structures, and improving the level of passenger and cargo transportation services.

## Data Availability

The datasets generated during and/or analyzed during the current study are available from the Official Airline Guide (https://oag.cn/).
